# Water Contamination Risks at the Dental Clinic

**DOI:** 10.3390/biology9030043

**Published:** 2020-02-27

**Authors:** Marco Cicciù

**Affiliations:** Department of Biomedical and Dental Sciences, Morphological and Functional Images, University of Messina, Policlinico G. Martino, Via Consolare Valeria, 98100 Me, Italy; acromarco@yahoo.it

**Keywords:** dentistry, water, infections, cross-infections, contamination, water contamination, risk

## Abstract

Dental clinics, furnished with an array of specialized equipment, are commonplace, particularly in industrialized countries. Minimizing the risk of infection at the dental practice requires the formulation and implementation of strict protocols. These protocols must address the real risk posed by water contamination, particularly given that water is both integral to the function of some dental equipment, and is typically administered directly to the patient. The water in the dental clinic may be of local origin or from a water main, this can be problematic since the clinician often has little assurance regarding the quality of water reaching the dental chair. Though most modern dental equipment includes self-sterilization protocols, care must be taken that water does not stagnate anywhere in the dental equipment or clinic. The management of water quality at the dental clinic is an important part of respecting the protocols needed to manage the risk of patient infections.

The prevention of infections in the dental office is a challenge that must be faced by every clinician. The culture and attention toward this important part of health care is increasing, driven in part by patient’s increasing concepts of their own personal protection. Infections can occur through the use of infected instruments, due to poor air quality, or the use of contaminated water. There is considerable (and justifiable) attention to instrument sterilization; but there is typically less attention to the treatment of air and water [1,2,3,4,5]. Increasingly, water has been identified as the cause of biological risk in the dental practice. Water is the vehicle through which most of the infections that develop in the dental office are spread. A biofilm that forms inside the piping of a building could contaminate the entire water network of that building, including a dental clinic. Within the dental clinic water spreads as an aerosol, increasing both bacterial spread within the clinic and the risk of contamination of all people in this area [6,7,8,9]. Various microorganisms such as single-cell algae, bacteria, and fungi have the potential to cover and infiltrate virtually every material in the dental clinic. Biofilms in dental clinics have been shown to include a dangerous contaminant bacterial deposit, which can become resistant to various disinfectants [9,10,11]. Biofilms within the water circuits of dental clinics, originate from one of two possible sources of contamination: from the public water supply, or from the patient’s mouth [11].

The public water supply should have a low bacterial load, with a total absence of pathogenic bacteria. This does not mean that the water is sterile. It will almost certainly contain a varied microbial flora which, by type and concentration, is generally harmless to humans. However, in particular conditions, pathogenic microorganisms or opportunistic pathogens can reach the dental unit from the water supply. It should be emphasized that the bacterial load of environmental waters is controlled at the origin by microbiological checks on samples taken upstream of the catchment area. However, the microbiological quality of the water recorded “at source” by the dispensing body does not necessarily correspond to the microbiological quality that could be detected at sites closer to the dental clinic [12,13,14,15,16,17,18,19].

When considering the risk of reflux water or dental instruments, it is necessary to consider all water-emitting tools. Turbines are instruments that have high rotation speeds and, in the slowdown phase, they have a centrifugal suction effect which makes them incorporate organic material from the tip of the instrument. For this reason, the rotary instruments are equipped with special valves, which should retain the aspirated material in the rotor. But these valves may not form an absolute seal, and any leakage can result in colonies of bacteria inside the unit. Since this is an ideal environment for bacterial growth, this can create a dangerous risk of infection; this is why all modern dental clinics have integrated disinfection systems [20,21,22,23,24,25,26].

Among the many infectious and pathogenic agents, both viral and bacterial, found in the water networks of dental clinics, there are particularly dangerous ones, including: legionella, pseudomonas, tuberculosis bacteria, HIV and hepatitis C viruses. Many of these could be verified through a simple bacteriological analysis of water. It must be borne in mind that through the instruments of the dental practice, viruses and bacteria present in the water supply are sprayed into the environment in the form of aerosols and come into direct contact with the wounds of the patient undergoing treatment. Furthermore, some patients of the dental practice are more susceptible to this route of infection given their hygienic and dental conditions [27,28,29,30,31,32,33,34,35,36]. 

There are different devices available to the dental clinic, which make it possible to limit the infection risk posed by water. There are devices that produce osmotic water and have a chlorine dioxide misting system inside the water, which constantly monitors and eliminates any presence of pathogens in the entire water circuit, including the dental clinic. All this happens automatically, without machine downtime, without making inquisitive actions and without human activity [37,38,39].

Chlorine dioxide is a powerful and effective disinfectant agent. Chlorine has been, and continues to be, a very effective disinfectant capable of making and maintaining safe drinking water. Chlorine dioxide works through an oxidative rather than chlorinate reaction. This virtually eliminates the formation of chlorinated organic compounds that are suspected to increase the risk of cancer [1,40,41]. The benefits of chlorine dioxide include:

1. Bactericidal efficiency is relatively unchanged at pH values between 4 and 10
2. It is effective in the destruction of spores, bacteria, viruses and other pathogenic organisms
3. It has high solubility
4. No corrosion is associated with the recommended dosages
5. Maintenance costs are reduced
6. It destroys phenols and has no distinctive odor
7. It removes iron and magnesium compounds better than chlorine, especially if they have complex bonds [1,42].

Reverse osmosis is the safest and most widespread water treatment system in the world, and is capable of guaranteeing the absolute purity of the water. The osmotic membrane is able to treat water at the molecular size, making it with optimal characteristics with regards to color, smell and organoleptic purity. Osmosis is a natural process that regulates the exchange of liquids. When two solutions with different salt concentration are separated from each other by a semipermeable membrane, which allows only the passage of the solvent and not of the solutes, the solvent of the most diluted solution will pass through the membrane in order to dilute the more concentrated one. By applying pressure on the surface of a concentrated solution, the natural flow of the liquid is reversed, thus obtaining a solution very poor in solutes from a highly concentrated starting liquid [43,44,45,46,47,48]. Other techniques for water purification are plasma ionization, or chemical products like chlorhexidine [47,49,50,51,52,53,54,55,56,57,58,59].

The risk of cross-infection between dentist and patient, or between patients, is high and consequently needs to be counteracted by appropriate, proven systems. The recent current interest in *Legionella pneumophila* pathologies must also make us reflect on other, much more worrying diseases and consequently pay attention to the adoption of systems capable of preventing them [3,9,20,26,37]. Patients in healthcare facilities are constantly exposed to infectious risks; understanding these risks, and constantly striving to minimize them, must be the goal of all clinicians.
